# Complete Genome Sequence of *Bacillus altitudinis* Type Strain SGAir0031 Isolated from Tropical Air Collected in Singapore

**DOI:** 10.1128/genomeA.01260-17

**Published:** 2017-11-09

**Authors:** Vineeth Kodengil Vettath, Ana Carolina M. Junqueira, Akira Uchida, Rikky W. Purbojati, James N. I. Houghton, Caroline Chénard, Daniela I. Drautz-Moses, Anthony Wong, Sandra Kolundžija, Megan E. Clare, Kenny J. X. Lau, Nicolas E. Gaultier, Cassie E. Heinle, Balakrishnan N. V. Premkrishnan, Elena S. Gusareva, Enzo Acerbi, Liang Yang, Stephan C. Schuster

**Affiliations:** a Singapore Centre for Environmental Life Sciences Engineering, Nanyang Technological University, Singapore; b Asian School of the Environment, Nanyang Technological University, Singapore

## Abstract

*Bacillus altitudinis* strain SGAir0031 (*Firmicutes*) was isolated from tropical air samples collected in Singapore. Its genome was assembled using short reads and single-molecule real-time sequencing, comprising one chromosome with 3.81 Mb and one plasmid with 32 kb. The genome consists of 3,820 protein-coding genes, 81 tRNAs, and 24 rRNAs.

## GENOME ANNOUNCEMENT

*Bacillus altitudinis* is a Gram-positive, rod-shaped aerobic bacterium classified in the phylum *Firmicutes*. It was first reported to be isolated from extreme UV-stressed air samples collected in the stratosphere (1). Since then, *B. altitudinis* has been reported in diverse habitats, including the southern Indian Ocean (2), deep freshwater of Manasbal Lake (3), soil (4), and silt (5).

*B. altitudinis* strain SGAir0031 was isolated from an air sample collected in Singapore (global position system [GPS] coordinates 1.346N, 103.680E) using the Andersen single-stage impactor (SKC BioStage) with a median cutoff diameter of 0.6 μm. The air was impacted onto Trypticase soy agar (TSA) (Becton Dickinson), and further isolation of colonies was carried out by culturing on TSA at 30°C and in Luria-Bertani (LB) broth overnight. Genomic DNA was purified using the Wizard genomic DNA purification kit (Promega), according to the manufacturer’s protocol, with an additional RNase digestion followed by phenol-chloroform cleanup and a final precipitation with isopropanol. Single-molecule real-time (SMRT) sequencing was performed on a PacBio RSII (Pacific Biosciences), using a concentration of 0.15 nM of the 8-kb genomic library loaded into one SMRT cell (Pacific Biosciences). Short reads were generated on a MiSeq (Illumina) 300-bp paired-end run using whole-genome shotgun libraries constructed with the TruSeq Nano DNA library preparation kit.

A total of 110,325 subreads were used for *de novo* assembly with Hierarchical Genome Assembly Process (HGAP) version 3 (6) implemented in the PacBio SMRT Analysis 2.3.0 package. The assembly was polished using Quiver (6) and error corrected using Pilon version 1.16 (7) and 836,357 MiSeq paired-end reads. The consensus assembly generated two contigs, one chromosome with 3,812,576 bp (173.78-fold coverage) and one plasmid with 32,110 bp (244.41-fold coverage). The chromosomal contig showed a mean G+C content of 41.4%, while the plasmid pSGAir0031 had 37.5% G+C content. Both the genome size and G+C content were similar to those of other *B. altitudinis* assemblies available.

Taxonomic identification at the species level was performed using Phyla-AMPHORA (8). A total of 152 *Firmicutes*-specific marker genes were matched out of 168. In addition, average nucleotide identity (ANI) analysis, performed with MiSI (Microbial Species Identifier) (9), showed 98.81% identity to *B. altitudinis* strain DSM 26896.

The genome was annotated using NCBI’s Prokaryotic Genome Annotation Pipeline (PGAP) version 4.2 (10). A total of 3,982 genes were predicted with 3,820 protein-coding genes (PCGs), 24 rRNA operons (5S, 16S, and 23S rRNAs), 81 tRNAs, 5 noncoding RNAs, and 52 pseudogenes. The average G+C content of the plasmid pSGAir0031 is 37.5%, with 33 PCGs and no tRNA or rRNA genes. Functional annotation performed with Rapid Annotations using Subsystems Technology (RAST) (11–13) showed that most genes were associated with carbohydrate metabolism (439 genes) and amino acid and derivative metabolism (436 genes). The strain SGAir0031 potentially forms spores, since 120 genes were found to be related to dormancy and sporulation. Sporulation could be a potential mechanism for the dispersal and survival of *B. altitudinis* in tropical air.

### Accession number(s).

The complete genome sequences of *Bacillus altitudinis* SGAir0031 and the plasmid pSGAir0031 have been deposited in DDBJ/EMBL/GenBank under the accession numbers CP022319 and CP022320, respectively.
