# Correction for Vettath et al., “Complete Genome Sequence of Bacillus altitudinis Strain SGAir0031 Isolated from Tropical Air Collected in Singapore”

**DOI:** 10.1128/MRA.00734-19

**Published:** 2019-08-01

**Authors:** Vineeth Kodengil Vettath, Ana Carolina M. Junqueira, Akira Uchida, Rikky W. Purbojati, James N. I. Houghton, Caroline Chénard, Daniela I. Drautz-Moses, Anthony Wong, Sandra Kolundžija, Megan E. Clare, Kenny J. X. Lau, Nicolas E. Gaultier, Cassie E. Heinle, Balakrishnan N. V. Premkrishnan, Elena S. Gusareva, Enzo Acerbi, Liang Yang, Stephan C. Schuster

**Affiliations:** a Singapore Centre for Environmental Life Sciences Engineering, Nanyang Technological University, Singapore; b Asian School of the Environment, Nanyang Technological University, Singapore

## AUTHOR CORRECTION

Volume 5, no. 45, e01260-17, 2017, https://doi.org/10.1128/genomeA.01260-17. Page 1: The title should appear as given in this correction.

